# Faster Hypothermia Induced by Esophageal Cooling Improves Early Markers of Cardiac and Neurological Injury After Cardiac Arrest in Swine

**DOI:** 10.1161/JAHA.118.010283

**Published:** 2018-10-25

**Authors:** Jiefeng Xu, Xiaohong Jin, Qijiang Chen, Chunshuang Wu, Zilong Li, Guangju Zhou, Yongan Xu, Anyu Qian, Yulin Li, Mao Zhang

**Affiliations:** ^1^ Department of Emergency Medicine Second Affiliated Hospital Zhejiang University School of Medicine Hangzhou China; ^2^ Institute of Emergency Medicine Zhejiang University Hangzhou China; ^3^ Department of Emergency Medicine Yuyao People's Hospital Medical School of Ningbo University Ningbo China; ^4^ Department of Emergency Medicine The First People's Hospital of Wenling Taizhou China; ^5^ Department of Intensive Care Medicine The First Hospital of Ninghai Ningbo China

**Keywords:** cardiac arrest, cardiopulmonary resuscitation, esophageal cooling, organ protection, therapeutic hypothermia, Cardiopulmonary Resuscitation and Emergency Cardiac Care

## Abstract

**Background:**

After cardiopulmonary resuscitation, the protective effects of therapeutic hypothermia induced by conventional cooling are limited. Recently, esophageal cooling (EC) has been shown to be an effective, easily performed approach to induce therapeutic hypothermia. In this study we investigated the efficacy of EC and its effects on early markers of postresuscitation cardiac and neurological injury in a porcine model of cardiac arrest.

**Methods and Results:**

Thirty‐two male domestic swine were randomized into 4 groups: sham control, normothermia, surface cooling, and EC. Sham animals underwent the surgical preparation only. Ventricular fibrillation was induced and untreated for 8 minutes while defibrillation was attempted after 5 minutes of cardiopulmonary resuscitation. At 5 minutes after resuscitation, therapeutic hypothermia was induced by either EC or surface cooling to reach a target temperature of 33°C until 24 hours postresuscitation, followed by a rewarming rate of 1°C/h for 5 hours. The temperature was normally maintained in the control and normothermia groups. After resuscitation, a significantly faster decrease in blood temperature was observed in the EC group than in the surface cooling group (2.8±0.7°C/h versus 1.5±0.4°C/h; *P*<0.05). During the maintenance and rewarming phases the temperature was maintained at an even level between the 2 groups. Postresuscitation cardiac and neurological damage was significantly improved in the 2 hypothermic groups compared with the normothermia group; however, the protective effects were significantly greater in the EC group.

**Conclusions:**

In a porcine model of cardiac arrest, faster hypothermia successfully induced by EC was significantly better than conventional cooling in improving early markers of postresuscitation cardiac and neurological injury.

**Clinical Perspective**

**What Is New?**

- Esophageal cooling is more effective than conventional surface cooling in inducing therapeutic hypothermia.
- Faster hypothermia induced by esophageal cooling can further improve early markers of postresuscitation cardiac and neurological injury compared with conventional surface cooling.

**What Are the Clinical Implications?**

- This indicates that esophageal cooling may become a promising cooling method to implement therapeutic hypothermia in the clinical setting.

After spontaneous circulation has been restored from cardiac arrest (CA), the ensuing myocardial dysfunction and brain injury become the major causes of high mortality and morbidity in victims.^1^, ^2^ A series of studies have confirmed that therapeutic hypothermia (TH) provides potent protection from cardiac and neurological damage after cardiopulmonary resuscitation (CPR).^3^, ^4^ However, only 35.7% of out‐of‐hospital CAs and 41% of in‐hospital CAs had favorable outcomes following hypothermic treatment.^5^, ^6^ Recently, another 2 investigations demonstrated that no differences in outcomes were observed between 33°C hypothermia and 36°C normothermia in CA victims.^7^, ^8^ The implementation strategy of TH urgently needs to be optimized.

Current opinions suggest that early and rapid induction, stable maintenance, and slow rewarming are key to exerting the protective effects of TH.^3^, ^9^, ^10^ However, it is difficult to achieve these requirements by the existing cooling techniques. Cold fluid infusion was effective to induce fast hypothermia, but it failed to improve the rate of return of spontaneous circulation (ROSC), survival, and neurological status.^11^, ^12^ Surface blanket cooling was feasible for the maintenance of mild hypothermia and subsequent rewarming; however, its lower cooling efficacy might lessen postresuscitation organ protection.^13^ Some novel cooling techniques including total liquid ventilation and continuous automated peritoneal lavage were shown to induce ultrafast hypothermia and to yield better outcomes.^4^, ^14^ However, these techniques have several limitations such as their invasive and complicated procedures and need for expensive and special equipment.

Recently, cooling via the esophagus was shown in some preliminary studies to be an effective approach to induction of TH.^15^, ^16^ Because esophageal cooling (EC) is a minimally invasive, low‐cost, and easily performed technique, it may provide a promising cooling method for the clinic. However, it remains unknown whether EC can induce TH and produce organ protection better than conventional cooling. The present study was designed to investigate the efficacy of EC and its effects on early markers of postresuscitation cardiac and neurological damage. We hypothesized that EC would induce more effective TH and further improve early markers of postresuscitation cardiac and neurological injury when compared to surface cooling (SC) in an experimental swine model of CA.

## Materials and Methods

All animals received humane care in compliance with the “Principles of Laboratory Animal Care” formulated by the National Society for Medical Research and the *Guide for the Care and Use of Laboratory Animals* prepared by the Institute of Laboratory Animal Resources. Healthy male, white domestic swine, aged 4 to 6 months, weighing 36±2 kg, were supplied by Shanghai Jiagan Biotechnology Inc (Shanghai, China). The animals were fed under the conditions of standard atmospheric pressure, 12/12‐hour light/dark cycle, room temperature (20°C to 25°C), humidity (60% to 80%), closed cage, spontaneous water intake, regular feeding, regular cleaning and disinfection. This study was approved by the Institutional Animal Care and Use Committee of the Zhejiang University School of Medicine. The data, analytic methods, and study materials in this study will be made available to other researchers for purposes of reproducing the results or replicating the procedure by the corresponding author on reasonable request.

### Animal Preparation

All animals were fasted overnight except for free access to water. Anesthesia was initiated by intramuscular injection of ketamine (20 mg/kg) and completed by an intravenous injection of sodium pentobarbital (30 mg/kg). Thereafter, sodium pentobarbital (8 mg/[kg·h]) and fentanyl (2 μg/[kg·h]) were continuously infused to maintain anesthesia. A cuffed endotracheal tube was advanced into the trachea. The animals were ventilated with a volume‐controlled ventilator (SynoVent E5; Mindray, Shenzhen, China) with a tidal volume of 12 mL/kg, peak flow of 40 L/min, and FiO_2_ of 0.21. End‐tidal CO_2_ was monitored with a handheld ETCO_2_/SPO_2_ monitor (PMSH‐300, SunLife Science Inc, Shanghai, China) and maintained at 35 to 40 mm Hg by adjusting respiratory frequency. The conventional lead II ECG was monitored.

For the measurement of myocardial function, a 7F central venous catheter was inserted into the left external jugular vein for the injection of iced saline, and another 4F thermistor‐tipped arterial catheter was inserted into the left femoral artery, both of which were connected to the PiCCO Monitor system (PiCCOplus, Pulsion Medical Systems, Munich, Germany). For the measurement of arterial pressure, a fluid‐filled 8F catheter (Model 6523, C.R. Bard Inc, Salt Lake City, UT) was advanced from the right femoral artery into the thoracic aorta. For the measurements of right atrial pressure and blood temperature, a 7F pentalumen, thermodilution tipped catheter (Abbott Critical Care # 41216, Chicago, IL) was advanced from the right femoral vein into the right atrium. For inducing ventricular fibrillation (VF), a 5F pacing catheter (EP Technologies Inc, Mountain View, CA) was advanced from the right external jugular vein into the right ventricle. All catheters were intermittently flushed with saline containing 5 IU/mL bovine heparin, and their positions were confirmed by characteristic pressure morphology and with fluoroscopy. Tympanic temperature was measured by an ear thermometer (PRO 4000, Braun Co, Southborough, MA), and rectal temperature was monitored with a thermal probe connected to the Blanketrol III Hyper‐Hypothermia System (Cincinnati Sub‐Zero, Cincinnati, OH). Body temperature was maintained at 38.0±0.5°C in all animals.

### Experimental Procedures

**Figure 1:**
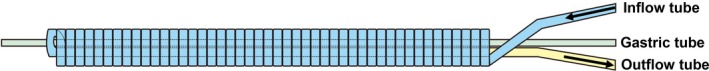
The design of the esophageal cooling device.

Ten minutes before induction of VF, baseline measurements were obtained. The animals were then randomized with the sealed envelope method into 1 of the 4 groups: (1) sham control (Control, n=5), (2) normothermia (n=9), (3) SC (n=9), or (4) EC (n=9). In the Control and normothermia groups a normal temperature of 38.0±0.5°C was maintained with the aid of Blanketrol III throughout the experiment. At 5 minutes after successful resuscitation, TH was induced in the 2 hypothermic groups to reach a targeted temperature of 33°C until 24 hours postresuscitation, followed by a rewarming rate of 1°C/h for 5 hours. SC was performed by surface blanket cooling, and EC was implemented by a novel EC device, both of which were connected to the Blanketrol Hyper‐Hypothermia System. The EC device was made of medical‐grade silicone with a length of 50 cm and diameter of 20 mm and consisted of 1 central gastric tube and 2 water‐circulating tubes (Figure 1). The inflow tube twined around the gastric and outflow tubes, and it circulated cold or warm water with the outflow tube in a closed circuit. The EC device was placed in the esophagus, and its placement was similar to that of a standard orogastric tube.

**Figure 2:**
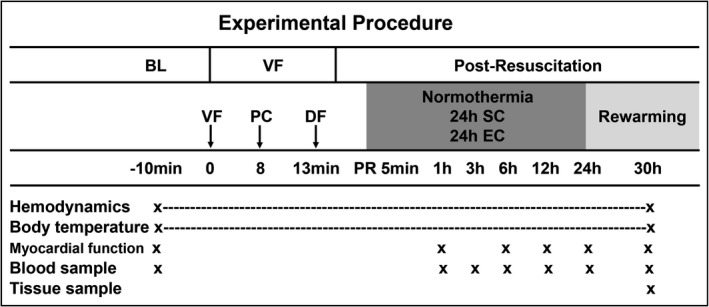
Experimental outline and procedure. BL indicates baseline; DF, defibrillation; EC, esophageal cooling; PC, precordial compression; PR, post resuscitation; SC, surface cooling; VF, ventricular fibrillation.

Sham animals underwent the surgical preparation only. In the other groups VF was induced by 1‐mA alternating current delivered to the right ventricular endocardium. Mechanical ventilation was discontinued after onset of VF. Before initiation of the resuscitation protocol, the pacing catheter was withdrawn. After 8 minutes of untreated VF, CPR was manually performed by a ratio of 30:2 of compression to ventilation. The compression quality was monitored by an E Series Monitor Defibrillator (ZOLL Medical Corporation, Chelmsford, MA) to guarantee a compression depth of 50 to 60 mm at a rate of 100 to 120 per minute. The ventilation was performed by a bag respirator attached to the endotracheal tube. After 2.5 minutes of CPR, a loading dose of 20 μg/kg of epinephrine was administered. After 5 minutes of CPR, defibrillation was attempted with a single 150‐J electrical shock delivered by the E Series Monitor Defibrillator. If an organized rhythm with a mean arterial pressure >50 mm Hg persisted for 5 minutes, the animal was regarded as ROSC. With failure to achieve ROSC, manual CPR was immediately resumed for 2 minutes before another defibrillation. This protocol was repeated until successful resuscitation or for a maximum of 10 minutes. The same dose of epinephrine was administered at an interval of 3 minutes if needed. When a recurrent VF occurred after resuscitation, a 150‐J electrical shock was attempted. Following ROSC, mechanical ventilation was continued with an FiO_2_ of 0.21 for 30 hours. Compound sodium chloride solution (2 mL/[kg·h]) was infused to maintain fluid balance, cisatracurium besylate (0.2 mg/[kg·h]) was intravenously administered to avoid shivering, and antibiotics (amoxicillin sodium and clavulanate potassium, 1.2 g) were intramuscularly injected every 12 hours to prevent infection. At 30 hours after resuscitation, the animals were euthanized with an intravenous injection of 150 mg/kg of sodium pentobarbital. The experimental outline is summarized in Figure 2.

### Measurements

Hemodynamics, ECG, and blood temperature were continuously recorded by a patient‐monitoring system (BeneView T6; Mindray, Shenzhen, China). Coronary perfusion pressure was calculated as the difference between decompression diastolic aortic and time‐coincident right atrial pressures. Tympanic and rectal temperatures were detected by an ear thermometer and a thermal probe, respectively. Blood gas and lactate concentrations were measured at baseline and 1, 3, 6, 12, 24, and 30 hours after resuscitation on 1.5‐mL arterial blood samples with a blood gas/electrolyte analyzer (Model 5700, Instrumentation Laboratory, Lexington, MA).

Global ejection fraction, as the indicator of myocardial contractile function, was measured with the PiCCO system at baseline and 1, 6, 12, 24, and 30 hours after resuscitation. Venous blood samples were collected at the same time points, and then the serum samples were obtained for the measurements of cardiac troponin I, neuron‐specific enolase, and S100B protein with enzyme‐linked immunosorbent assay kits (Meixuan Biotechnology Inc, Shanghai, China) according to the manufacturer's instructions.

Both left ventricular myocardium and right frontal lobe were harvested for the evaluation of inflammatory and oxidative injuries at 30 hours postresuscitation. The specimens were homogenized with normal saline on ice and centrifuged at 1600 g at 4°C for 15 minutes. The supernatants were then collected for measuring the levels of tumor necrosis factor‐α and interleukin‐6 with enzyme‐linked immunosorbent assay kits (Meixuan Biotechnology Inc, Shanghai, China). The levels of malondialdehyde were determined by the thiobarbituric acid–reactive substances assay, and the activities of superoxide dismutase were determined by the xanthine oxide assay based on the previous methods.^17^ The assay kits of malondialdehyde and superoxide dismutase were purchased from Nanjing Jiancheng Bioengineering Institute (Nanjing, China). The results were expressed as per microgram of protein.

In addition, left ventricular myocardium and left frontal lobe were harvested to measure cell apoptosis and cleaved caspase‐3 expression in the heart and brain. All the specimens were fixed in 4% paraformaldehyde for 24 hours, then embedded in paraffin, and finally cut in a 5‐μm section. For the measurement of cell apoptosis, the sections were stained using a TdT‐mediated dUTP nick end labeling assay kit (Roche Diagnostics GmbH, Mannheim, Germany) according to the manufacturer's instructions, and then 6 views were randomly chosen to count the numbers of TdT‐mediated dUTP nick end labeling–positive cells and total cells at ×200 magnification under an optical microscope (Biological Microscope CX31, Olympus, Tokyo, Japan). The rate of apoptosis in cells was calculated as the percentage of TdT‐mediated dUTP nick end labeling–positive cells/total cells. For the measurement of cleaved caspase‐3 expression, the sections were incubated with primary anti–cleaved caspase‐3 (1:200; Cell Signaling Technology Inc, Danvers, MA), then treated with the secondary antibody, and finally reacted with diaminobenzidine (Boster Biological Technology Ltd, Wuhan, China). Similarly, 6 images of immunohistochemical staining were randomly captured under ×200 magnification. The semiquantitative analysis of the intensity of cleaved caspase‐3‐positive staining was performed through integrated optical density using Image‐Pro Plus 6.0 software (Media Cybernetics, Silver Spring, MD) according to the method of Xing et al.^18^

Tissue of the lower esophagus was also harvested for pathological analysis at 30 hours postresuscitation. After the specimen was processed using the above‐mentioned method, the section was stained with hematoxylin and eosin. Photomicrographs were taken and then examined under ×200 magnification.

### Statistical Analysis

Data were analyzed using SPSS version 18.0 (IBM, Armonk, NY). Continuous variables were presented as mean±standard deviation when data were normally distributed or as a median (25th, 75th percentiles) when data were not normally distributed. Normal distribution was confirmed with the Kolmogorov‐Smirnov test. Variables were compared with 1‐way analysis of variance or the Kruskal‐Wallis test for nonparametric data. Comparisons between time‐based measurements within each group were performed with repeated‐measurement analysis of variance. If there was a significant difference in the overall comparison of groups, comparisons between any other 2 groups were made by the Bonferroni test. For the comparison of categorical variables such as ROSC, the Fisher exact test was used. A value of *P*<0.05 was considered significant.

## Results

Thirty‐two studies were performed and completed. There were no differences in baseline hemodynamics, myocardial function, arterial lactate, or blood temperature among the 4 groups (Table 1). During CPR, the same level of coronary perfusion pressure was achieved, and no difference was observed among the normothermia, SC, and EC groups. Consequently, 8 of the 9 animals were successfully resuscitated in each group. The same CPR outcomes including ROSC, duration of CPR, number of defibrillations, and total dosage of epinephrine were obtained in the 3 groups (Table 2).

**Table 1:** Baseline Characteristics

Variables	Control	Normothermia	SC	EC
Body weight, kg	36.2±3.0	36.7±2.4	36.1±2.9	36.7±2.1
Heart rate, beats/min	104±5	107±12	107±11	105±11
Mean arterial pressure, mm Hg	120±8	119±12	115±12	119±11
End‐tidal CO_2_, mm Hg	40±2	40±3	39±3	40±2
Global ejection fraction, %	31.6±1.3	30.6±4.1	31.1±3.0	30.0±4.2
pH	7.46±0.10	7.46±0.02	7.49±0.03	7.47±0.03
Arterial lactate, mmol/L	1.2±0.7	1.2±0.4	1.3±0.4	1.1±0.3
Blood temperature, °C	37.9±0.4	38.0±0.3	37.9±0.3	37.9±0.4

Values are presented as mean±SD. EC indicates esophageal cooling; SC, surface cooling.

**Table 2:** Coronary Perfusion Pressure, Return of Spontaneous Circulation, Duration of Cardiopulmonary Resuscitation, Number of Defibrillations, and Dosage of Epinephrine

Variables	Normothermia	SC	EC
CPP in PC1, mm Hg	17.3±3.0	17.6±2.5	18.6±3.8
CPP in PC3, mm Hg	28.1±3.2	29.1±5.0	30.3±5.1
CPP in PC5, mm Hg	26.4±4.3	26.8±5.8	25.3±4.4
ROSC, n/n	8/9	8/9	8/9
Duration of CPR, min	5.0 (5.0, 7.0)	5.0 (5.0, 5.0)	5.0 (5.0, 5.0)
Number of defibrillations, n	1.0 (1.0, 2.0)	1.0 (1.0, 1.0)	1.0 (1.0, 1.0)
Dosage of epinephrine, mg	0.82 (0.69, 1.48)	0.72 (0.65, 0.81)	0.76 (0.72, 0.78)

Values are presented as mean±SD or median (25th, 75th percentiles). CPP indicates coronary perfusion pressure; CPR, cardiopulmonary resuscitation; EC, esophageal cooling; PC1, 1 minute after precordial compression; PC3, 3 minutes after precordial compression; PC5, 5 minutes after precordial compression; ROSC, return of spontaneous circulation; SC, surface cooling.

**Figure 3:**
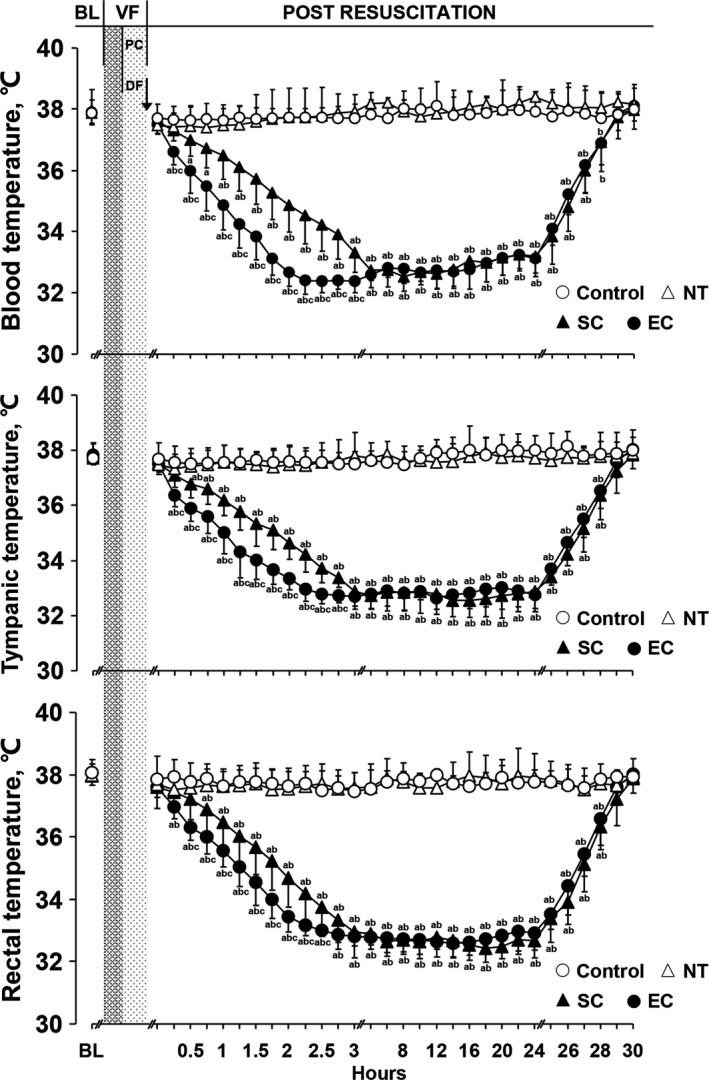
The changes of blood, tympanic, and rectal temperatures in the different groups (note: except the control group containing 5 swine, the other groups have 8 swine each). ^a^ *P*<0.05 vs Control group, ^b^ *P*<0.05 vs NT group, ^c^ *P*<0.05 vs SC group. The bar length represents the standard deviation. BL indicates baseline; DF, defibrillation; EC, esophageal cooling; NT, normothermia; PC, precordial compression; SC, surface cooling; VF, ventricular fibrillation.

After resuscitation, blood, tympanic, and rectal temperatures were maintained at a normal level in the Control and normothermia groups. However, these temperatures were rapidly decreased in the 2 hypothermic groups, in which the cooling rates were significantly faster in the EC group than in the SC group. The time to target temperature was significantly shortened in the animals receiving EC compared to the SC group. During the maintenance and rewarming phases, these temperatures were maintained at an even level, and no differences were observed between the 2 hypothermic groups (Figure 3, Table 3).

**Table 3:** Efficacy of Temperature Control in the Hypothermic Groups

Variables	Blood Temperature		Tympanic Temperature		Rectal Temperature	
	SC	EC	SC	EC	SC	EC
Induction phase						
Cooling rate, °C/h	1.5±0.4	2.8±0.7^a^	1.6±0.1	2.2±0.3^a^	1.7±0.3	2.1±0.3^a^
Time to target temperature, min	185±35	102±17^a^	169±12	128±18^a^	169±36	135±18^a^
Maintenance phase						
Maximum temperature, °C	33.7±0.3	33.7±0.3	33.4±0.5	33.5±0.5	33.3±0.2	33.5±0.4
Minimum temperature, °C	32.2±0.2	32.0±0.2	32.1±0.4	32.3±0.3	32.1±0.2	32.2±0.4
Mean temperature, °C	32.9±0.2	32.7±0.2	32.7±0.4	32.8±0.4	32.6±0.2	32.8±0.3
Temperature variability, °C	1.6±0.4	1.7±0.2	1.4±0.3	1.1±0.4	1.2±0.3	1.3±0.5
Rewarming phase						
Rewarming rate, °C/h	0.9±0.1	0.9±0.1	0.9±0.2	1.0±0.2	0.9±0.2	0.9±0.1

Values are presented as mean±SD. Temperature variability was calculated as difference between maximum and minimal temperature. EC indicates esophageal cooling; SC, surface cooling.

a *P*<0.05 vs SC group.

**Figure 4:**
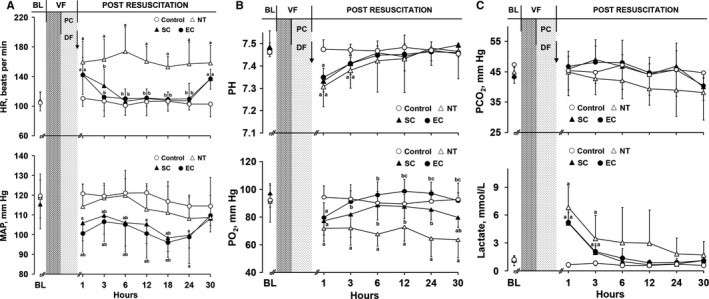
The changes of hemodynamics, blood gases, and lactate in the different groups (note: the control group contained 5 swine; the other groups had 8 swine each). A, Heart rate (HR) and mean arterial pressure (MAP). B, Arterial pH and po_2_. C, Arterial pco_2_ and lactate. ^a^ *P*<0.05 vs Control group, ^b^ *P*<0.05 vs NT group, ^c^ *P*<0.05 vs SC group. The bar length represents the standard deviation. BL indicates baseline; DF, defibrillation; EC, esophageal cooling; NT, normothermia; PC, precordial compression; SC, surface cooling; VF, ventricular fibrillation.

After resuscitation, heart rate was increased, and mean arterial pressure was decreased, in the 2 hypothermic groups; however, heart rate rapidly decreased to a near‐baseline level and mean arterial pressure was stably maintained at a normal physiological level during hypothermic treatment (Figure 4A). After resuscitation, pH and po_2_ were decreased, and lactate was increased, in the normothermia, SC, and EC groups. However, pH and lactate returned to near‐baseline levels starting 6 hours after resuscitation in the 3 groups. Nevertheless, the improvement in po_2_ was just observed in the 2 hypothermic groups, in which the values were significantly greater than that in the normothermia group starting 6 hours after resuscitation. No difference was observed in arterial pco_2_ among the 3 groups (Figure 4B and 4C).

**Figure 5:**
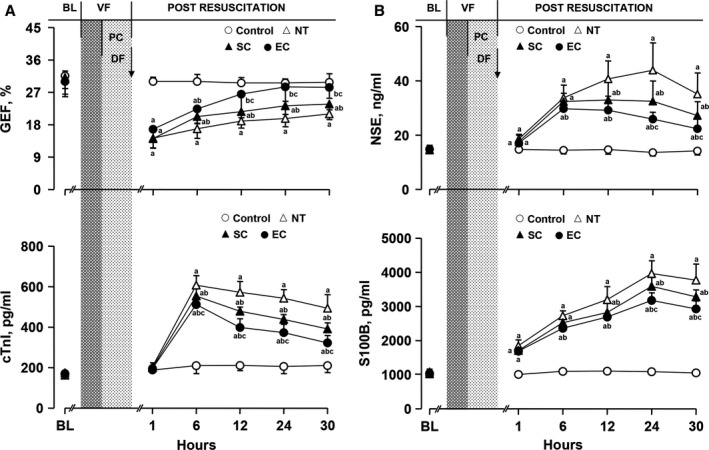
Changes of myocardial function and serum cardiac and cerebral biomarkers in the different groups (note: the control group contained 5 swine; the other groups had 8 swine each). A, Global ejection fraction (GEF) and serum cardiac troponin I (cTnI). B, Serum neuron‐specific enolase (NSE) and S100B protein (S100B). ^a^ *P*<0.05 vs Control group, ^b^ *P*<0.05 vs NT group, ^c^ *P*<0.05 vs SC group. The bar length represents the standard deviation. BL indicates baseline; DF, defibrillation; EC, esophageal cooling; NT, normothermia; PC, precordial compression; SC, surface cooling; VF, ventricular fibrillation.

After resuscitation, global ejection fraction was decreased, and serum cardiac troponin I, neuron‐specific enolase, and S100B protein were increased, in the normothermia, SC, and EC groups. However, the values of global ejection fraction were significantly increased, and the serum levels of cardiac troponin I were significantly decreased, starting 6 hours after resuscitation in the 2 hypothermic groups when compared with the normothermia group. Both of them were further significantly improved starting 12 hours after resuscitation in the EC group compared to the SC group (Figure 5A). The serum levels of neuron‐specific enolase and S100B protein were significantly lower starting 12 hours after resuscitation in the 2 hypothermic groups than in the normothermia group. Both of them were further significantly decreased starting 24 hours after resuscitation in the EC group compared to the SC group (Figure 5B).

**Figure 6:**
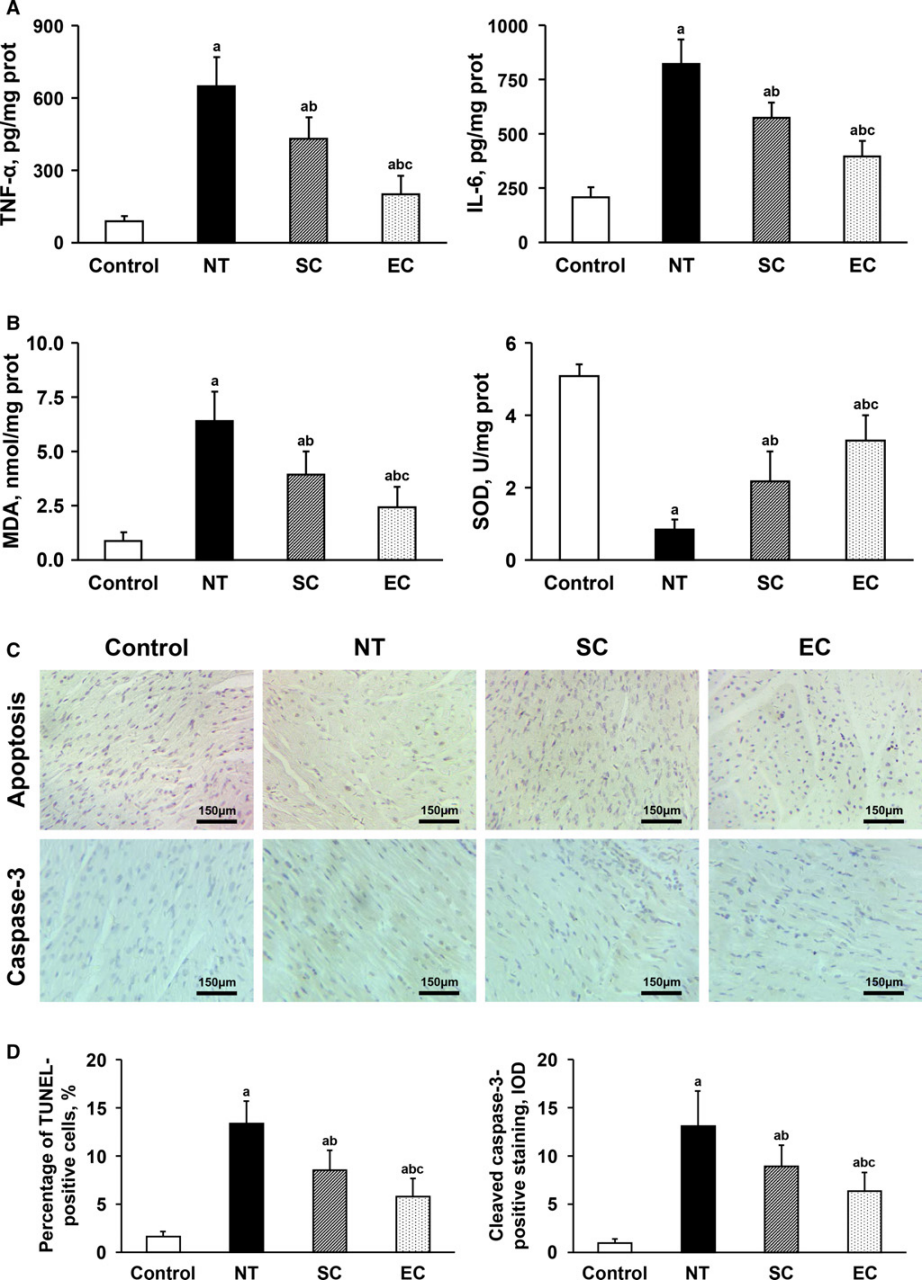
Comparisons of tissue inflammation, oxidative stress, and cell apoptosis in the heart among the 4 groups (note: the control group contained 5 swine; the other groups had 8 swine each). A, Tumor necrosis factor‐α (TNF‐α) and interleukin‐6 (IL‐6). B, Malondialdehyde (MDA) and superoxide dismutase (SOD). C, Representative photomicrographs of TdT‐mediated dUTP nick end labeling (TUNEL) assay and cleaved caspase‐3 immunostaining (×200 magnification). D, The percentage of TUNEL‐positive cells and the integrated optical density (IOD) values of cleaved caspase‐3–positive staining. ^a^ *P*<0.05 vs Control group, ^b^ *P*<0.05 vs NT group, ^c^ *P*<0.05 vs SC group. The bar length represents the standard deviation. EC indicates esophageal cooling; NT, normothermia; SC, surface cooling.

**Figure 7:**
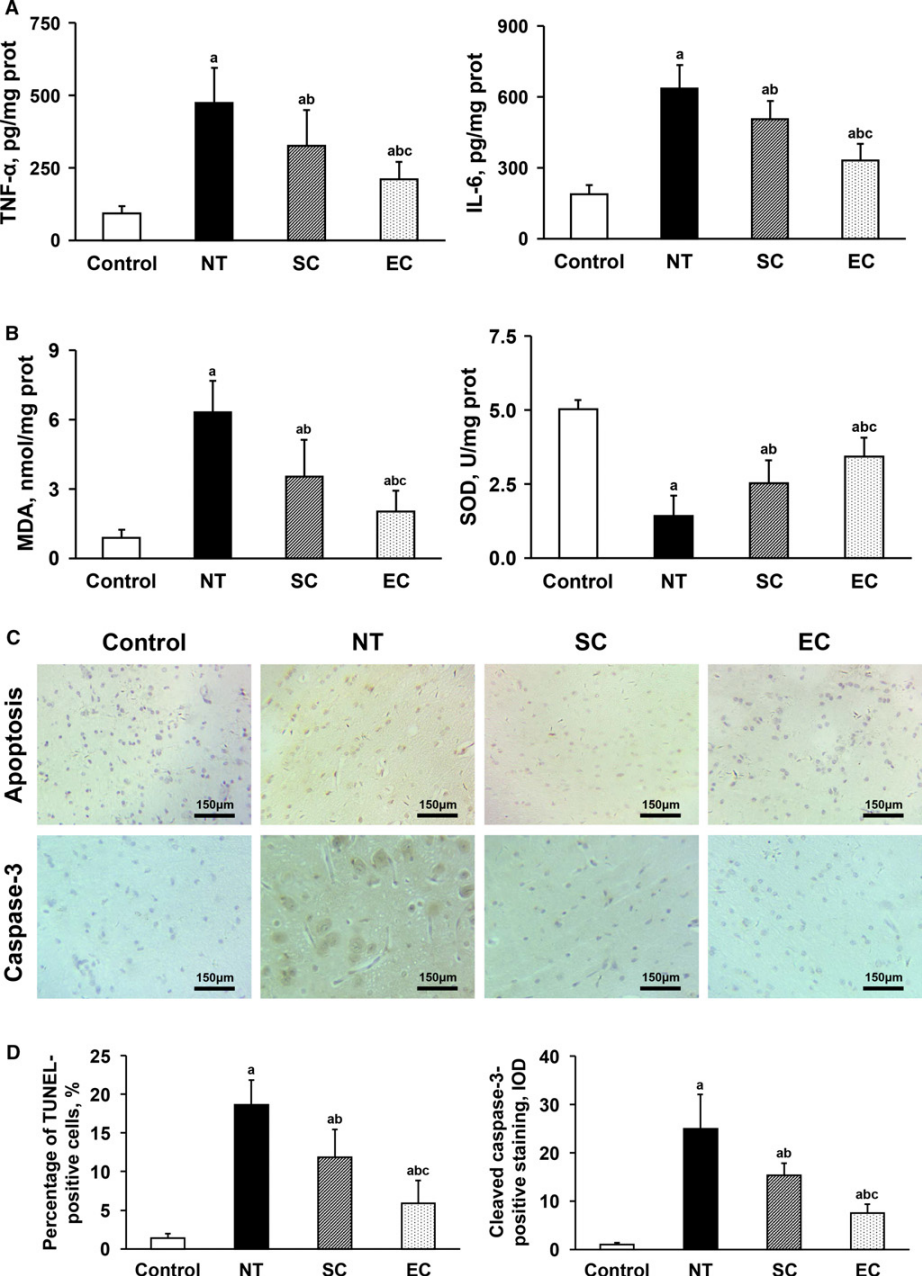
Comparisons of tissue inflammation, oxidative stress, and cell apoptosis in the brain among the 4 groups (note: the control group contained 5 swine; the other groups had 8 swine each). A, Tumor necrosis factor‐α (TNF‐α) and interleukin‐6 (IL‐6). B, Malondialdehyde (MDA) and superoxide dismutase (SOD). C, Representative photomicrographs of TdT‐mediated dUTP nick end labeling (TUNEL) assay and cleaved caspase‐3 immunostaining (×200 magnification). D, The percentage of TUNEL‐positive cells and the integrated optical density (IOD) values of cleaved caspase‐3–positive staining. ^a^ *P*<0.05 vs Control group, ^b^ *P*<0.05 vs NT group, ^c^ *P*<0.05 vs SC group. The bar length represents the standard deviation. EC indicates esophageal cooling; NT, normothermia; SC, surface cooling.

At 30 hours postresuscitation, the levels of tumor necrosis factor‐α, interleukin‐6, and malondialdehyde were significantly increased, and the activities of superoxide dismutase were significantly decreased, in the heart and brain in the normothermia, SC, and EC groups when compared with the Control group. Likewise, the percentage of TdT‐mediated dUTP nick end labeling–positive cells and the integrated optical density values of cleaved caspase‐3–positive staining in the heart and brain were significantly increased in the 3 groups. However, tissue inflammation, oxidative stress, and cell apoptosis in the heart and brain were significantly milder in the 2 hypothermic groups than in the normothermia group. Additionally, all of these pathological injuries in the heart and brain were further significantly alleviated in animals treated with EC compared to the SC group (Figures 6 and 7).

**Figure 8:**
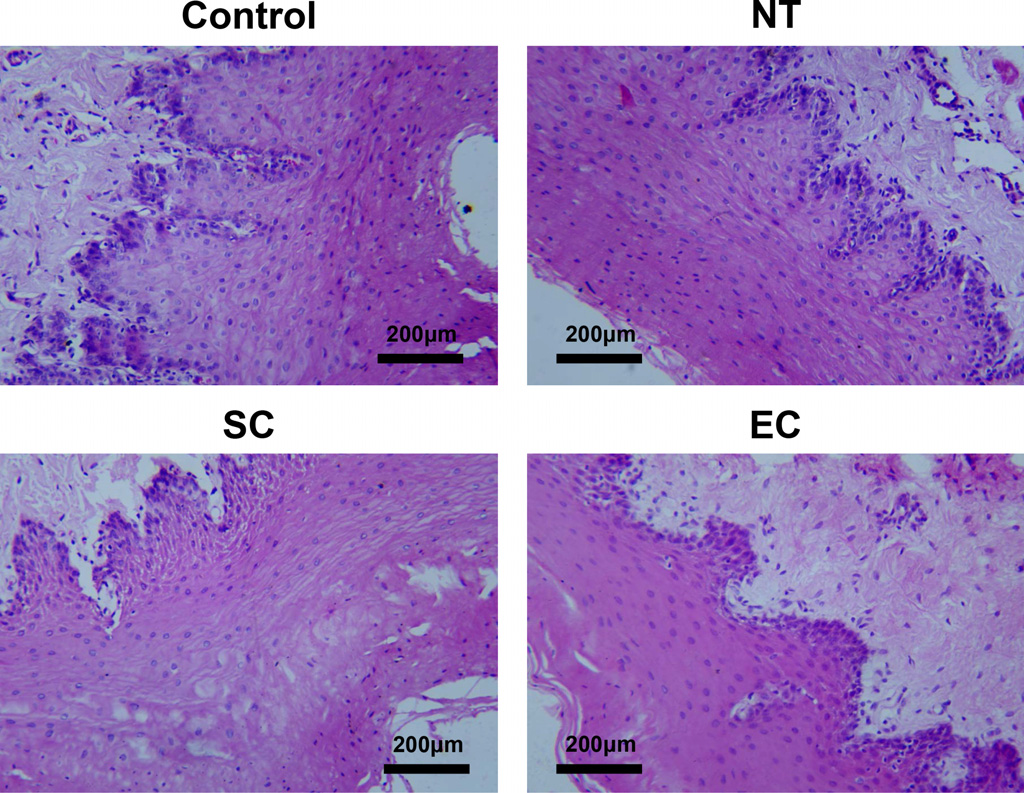
Representative photomicrographs of tissue pathology of lower esophagus in the different groups. EC indicates esophageal cooling; NT, normothermia; SC, surface cooling.

At 30 hours postresuscitation, mild inflammatory infiltrates were found in the lower esophagus in the normothermia, SC, and EC groups. No significant differences were observed among the 3 groups (Figure 8).

## Discussion

The present study demonstrated that EC was significantly better in the induction of TH and equally effective for its maintenance and rewarming when compared with conventional SC. No bolus of cold fluid was administered to assist the induction of hypothermia in all hypothermic animals. Consequently, early markers of postresuscitation cardiac and neurological damage were significantly alleviated in the 2 hypothermic groups compared to the normothermia group, in which the protective effects were related to the inhibition of tissue inflammation, oxidative stress, and cell apoptosis. However, faster hypothermia induced by EC produced significantly more potent cardiac and neurological protection compared with the SC group.

In 2012 Vaicys et al^19^ first demonstrated that the induction of TH via the esophagus was a feasible approach in a mathematical model. In theory the body heat can be rapidly extracted via the esophagus because its location is near to those organs and vessels with large volumes of blood flow, such as heart, lung, aorta, and vena cava. Subsequently, Kulstad et al^20^ developed an EC device with a diameter of 12 mm to verify its effectiveness in inducing TH in an adult swine model and demonstrated that a cooling rate of 1.3°C/h was achieved in swine with a mean weight of 65 kg. Recently, Schroeder et al^15^ compared 2 diameters (11 and 14.7 mm) of EC devices in pediatric swine averaging 27.2 kg and showed that they produced equally effective cooling at rates of 2.8°C/h and 3.0°C/h, respectively. However, all these experimental studies were performed under normal physiological conditions. The cooling efficacy of EC may be lessened in animals experiencing CA, as abnormal hemodynamics after resuscitation can limit heat exchange from the blood to the esophagus.^21^ Currently, the decrease in the effectiveness of EC has been observed in post‐CA care in 2 small observational clinical studies. One investigation demonstrated that EC in combination with iced fluid infusion achieved a mean cooling rate of 1.12°C/h^22^; however, another investigation demonstrated that the cooling rate was decreased to 0.26°C/h when using the EC alone.^16^ Thus, the effectiveness of EC remains to be further optimized.

In the present study a novel EC device was developed based on the previous studies.^15^, ^20^ First, a larger diameter of 20 mm was chosen to further increase the volume of water flow within the tube and also the heat‐exchange surface between the esophagus and blood. Second, the inflow tube was designed to twine around the outflow tube. It might be more beneficial to totally take heat energy out and avoid its returning to the body. Consequently, even in abnormal physiological conditions following CA and resuscitation, a cooling rate of 2.8°C/h in blood was achieved in swine averaging 36.7 kg in the EC group, which was approximately twice of the speed of cooling in the SC group. The rates of cooling in tympanum and rectum were also significantly greater in the EC group than in the SC group. Subsequently, the temperatures in these sites were equally maintained, followed by an even rewarming in both the EC and SC groups. It might be attributed to a reduced requirement for the efficacy of temperature regulation during the maintenance and rewarming phases. In addition, mild inflammatory injury was observed in the esophagus in all resuscitated animals. Thus, the esophageal damage might be mainly related to the injury resulting from CA and resuscitation but not to the pressure produced by a larger diameter of EC device. The EC might become an effective and safe cooling method better than conventional SC.

Currently, the main objective for optimizing the cooling technique is to improve postresuscitation multiple organ protection, especially cardiac and neurological outcomes. There has been increasing evidence that the protective effects of TH are closely associated with its induction, maintenance, and rewarming. In 2011 Chenoune et al^4^ demonstrated that ultrafast cooling with only 10 minutes to target temperature exerted postresuscitation cardiac and neurological protection, whereas the protection was not obvious when conventional cooling requiring 30 minutes to target temperature was used. In 2014 Suh et al^23^ demonstrated that the stable maintenance of 48 hours of TH was better than 24 hours of TH in alleviating postresuscitation neurological damage through the inhibition of brain apoptosis. Lu et al^3^ demonstrated that a rewarming rate of 0.5°C/h to 1°C/h did not alter postresuscitation cardiac and neurological protection produced by TH; however, a rewarming rate of 2°C/h abolished its protective effects. In this study significantly faster induction was achieved followed by the identical maintenance and rewarming in animals treated with EC compared to the SC group. Consequently, postresuscitation myocardial dysfunction and cardiac and cerebral injuries were significantly milder in the EC group than in the SC group, which was further confirmed by pathological evaluation indicating the decrease of tissue inflammation, oxidative stress, and cell apoptosis in these organs. Thus, significantly more potent protection produced by EC might be mainly attributed to a more rapid onset of TH and then a longer treatment duration. In any case, EC might provide a novel cooling method that facilitates postresuscitation cardiac and neurological outcomes.

### Limitations

There were several limitations in our study. First, a higher rate of 1°C/h was chosen to rewarm the animals. This rewarming rate does not alter the beneficial effects of TH based on our previous study.^3^ However, the patients should be rewarmed at a rate of 0.25°C/h to 0.5°C/h according to the current guidelines.^24^ Second, all animals were continuously monitored under anesthesia throughout the experiment and then euthanitized for the examination of tissue pathology. As a result, we failed to evaluate postresuscitation neurological function and survival. However, because CA and resuscitation represent a complex cascade of pathophysiological processes including systemic ischemia‐reperfusion response and multiple organ injury, a longer observation period is needed to fully evaluate those functional and pathological outcomes of vital organs so as to further confirm the protective effects of therapeutic interventions in the future. Third, only the lower esophagus was harvested for the evaluation of esophageal injury, which might not reflect the overall situation of esophageal damage. Fourth, because sodium pentobarbital has a longer half‐time and also neuroprotective properties, its continuous use might affect the outcomes of CA.

## Conclusions

In a porcine model of CA, EC enabled more rapid achievement of target temperature and significantly improved early markers of postresuscitation cardiac and neurological injury compared with conventional SC.

## Sources of Funding

This study was supported by the Natural Science Foundation of China (81571916), the Welfare Scientific Research Project from the Chinese Ministry of Health (2015SQ00050), the Zhejiang Provincial Natural Science Foundation of China (LY17H150001), the Zhejiang Provincial Welfare Scientific Research Project of China (LGF18H150003), the Zhejiang Provincial Medical Science Foundation of China (2018241256), and the Taizhou Civic Medical Science Foundation of China (1702KY57).

## Disclosures

None.
